# Emerging insights into CC and CXC chemokines and their receptors in *Mycobacterium tuberculosis* infection

**DOI:** 10.1002/2211-5463.70269

**Published:** 2026-05-12

**Authors:** Xuying Yin, Dangsheng Xiao, Jiezuan Yang

**Affiliations:** ^1^ Department of Nursing, the First Affiliated Hospital Zhejiang University School of Medicine Hangzhou China; ^2^ State Key Laboratory for Diagnosis and Treatment of Infectious Diseases, The First Affiliated Hospital, Zhejiang University School of Medicine National Clinical Research Center for Infectious Diseases Hangzhou China; ^3^ Zhejiang Provincial Key Laboratory for Diagnosis and Treatment of Aging and Physic‐chemical Injury Diseases, Department of Geriatrics, the First Affiliated Hospital Zhejiang University School of Medicine Hangzhou China

**Keywords:** biomarkers, chemokines, immune regulation, *Mycobacterium tuberculosis*, therapeutic targets

## Abstract

Chemokines and their cognate receptors are central orchestrators of the immune response to *Mycobacterium tuberculosis* (Mtb) infection. While their overall significance in tuberculosis (TB) is well‐established, this review synthesizes recent advances to clarify the distinct roles of CC and CXC chemokines in differentiating active disease, latent infection, and the often overlooked subclinical TB state. We evaluate the potential of specific chemokine signatures as emerging diagnostic biomarkers compared to conventional standards and assess their promise as novel therapeutic targets in personalized clinical settings. Furthermore, we examine paradoxical findings in the field, including how certain chemokines (such as CCL5, CXCL12, and CXCL16) can simultaneously support host defense and facilitate pathogen evasion. By integrating these complex narratives, we offer a renewed perspective on chemokine dynamics in TB immunity, bridge important gaps between bench research and clinical application, and establish a strong foundation for developing precision diagnostics and host‐directed therapies.

## Graphical abstract

The dual roles of CC and CXC chemokines in distinguishing active, latent, and subclinical tuberculosis were reviewed, along with an evaluation of their potential as diagnostic biomarkers and therapeutic targets to advance precision medicine in tuberculosis management. The graphical abstract was generated with AI assistance (Gemini 3.0).

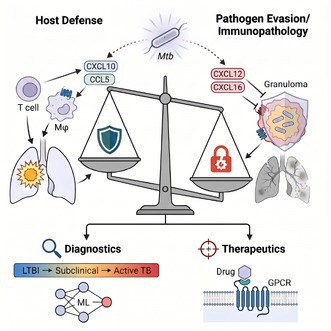

**Abbreviations**
ACKR1: Atypical chemokine receptor 1
AIDS: Human immunodeficiency syndrome
ATB: Active tuberculosis
BAL: Bronchoalveolar lavage fluid
BCA‐1: B Cell‐Attracting Chemokine‐1
BLC: B Lymphocyte chemoattractant
CCL2: C‐C motif chemokine ligand 2
CCR8: C‐C motif chemokine receptor 8
CRG2: Controlled by Retinoic Acid Gene 2
CXCL10: C‐X‐C motif chemokine ligand 10
CXCR3: C‐X‐C motif chemokine receptor 3
DCs: Dendritic cells
ELC: EBI1 ligand chemokine
ENA78: Epithelial‐derived Neutrophil‐activating peptide 78
GCP2: Granulocyte Chemotactic Protein‐2
GPCRs: G protein‐coupled receptors
GRO‐α: Growth‐regulated oncogene alpha
GRO‐β: Growth‐regulated oncogene beta
IFN‐γ: Interferon‐gamma
IL‐8: Interleukin‐8
IP‐10: Interferon gamma‐induced protein‐10
ITAC: Interferon‐inducible T‐cell alpha chemoattractant
LTBI: Latent tuberculosis infection
MCP‐1: Monocyte chemoattractant protein‐1
MGSA: Melanoma growth‐stimulating activity
MIG: Monokine induced by interferon‐gamma
MIP‐1α: Macrophage inflammatory protein‐1alpha
MIP‐2α: Macrophage Inflammatory Protein‐2alpha
Mtb: Mycobacterium tuberculosis
Mφ: Macrophage
NAP‐2: Neutrophil‐activating peptide‐2
NAP‐3: Neutrophil‐activating protein‐3
PBSF: Pre‐B cell growth‐stimulating factor
RANTES: Regulated on activation, normal T cell expressed and secreted
SCYA1: Small‐Inducible Cytokine A1
SCYB1: Small‐inducible cytokine B1
SDF‐1: Stromal cell‐derived factor‐1
SLC: Secondary lymphoid tissue chemokines
TNF‐α: Tumor necrosis factor‐alpha

Tuberculosis (TB), caused by *Mycobacterium tuberculosis* (Mtb), remains a global health problem. It is one of the leading causes of death worldwide, surpassing HIV/AIDS in mortality attributable to a single infectious agent. According to the World Health Organization (WHO), tuberculosis claimed 1.23 million lives in 2024. An estimated 10.7 million new tuberculosis cases were identified worldwide, including 8.3 million newly diagnosed cases [1]. Mtb is transmitted via aerosols and primarily infects alveolar macrophages, neutrophils, and dendritic cells (DCs) [2]. The immunopathogenesis of TB depends on a robust cellular response in which chemokine‐mediated recruitment of Th1 cells to the site of infection, driven by CXCR3 and its ligands, plays a vital role [3, 4]. In a mouse model, deletion of the murine immunity‐related GTPase M1 (IRGM1) ortholog increases IRGM3‐dependent type I IFN signaling, which prevents T‐cell expansion and eliminates T‐cell–mediated control of Mtb infection in neutrophils and alveolar macrophages [5]. Moreover, Curdlan enhances anti‐TB activity by regulating nitric oxide (NO) production through inducible NO synthase (iNOS) induction *in vitro* and in a mouse model, representing a promising host‐directed therapy. However, the inhibitory effect on human TB still requires further verification [6].

Despite advances in TB treatment, diagnostic challenges remain. Current methods, such as sputum smear microscopy, have low sensitivity, while Mtb culture, the diagnostic gold standard, is time‐consuming. IFN‐γ release assays (IGRAs), such as the T‐spot, are limited by high cost and variable specificity, which restricts widespread use [7]. Therefore, there is an urgent need for innovative, rapid, and cost‐effective diagnostic tools. Emerging evidence suggests that chemokines and their receptors are promising candidates for distinguishing between active TB and latent TB infection (LTBI), as well as for evaluating treatment efficacy [8, 9]. Furthermore, chemokines could enhance our ability to identify subclinical and difficult‐to‐diagnose TB [10]. Subclinical TB lies between LTBI and active TB [11].

Chemokines, a family of small secreted proteins, regulate immune cell migration by binding to G protein‐coupled receptors. The CC and CXC subfamilies are central to TB immunology, controlling granuloma formation and lymphocyte recruitment [12, 13]. Recent studies highlight their diagnostic potential, but gaps remain in translating these findings into clinical applications [14]. This review summarizes recent research on CC and CXC chemokines in TB immunity and presents three key innovations: (a) Diagnostic precision: We describe chemokine signatures that may outperform conventional biomarkers in distinguishing TB stages and propose a roadmap for noninvasive testing. (b) Therapeutic potential: By clarifying how chemokine receptor dynamics affect disease progression, we identify targets for immunomodulatory therapies. (c) Original findings: Unlike previous reviews, we critically evaluate conflicting evidence, including the dual roles of CXCL12 and CXCL16 in host defense and pathogenicity, and suggest future research directions.

Our analysis consolidates fragmented literature and advances the field by highlighting understudied chemokines. In a mouse model, one study shows that group 3 innate lymphoid cells (ILC3s) mediate early protective immunity against Mtb by inducing CXCL13 expression, identifying the CXCR5–CXCL13 axis as critical for Mtb control [15]. By addressing these gaps, this work aims to promote the development of next‐generation diagnostics and host‐specific therapies, ultimately bridging laboratory research and clinical application.

## Overview of chemokines

Chemokines are a highly conserved family of small (8–12 kDa) secreted proteins. Their ability to regulate leukocyte transport through G protein‐coupled receptors (GPCRs) highlights their importance in host defense, particularly against infections with Mtb [16]. Structurally, chemokines are classified into four subfamilies based on the arrangement of conserved N‐terminal cysteine residues: CC, CXC, C, and CX3C [17]. The CC and CXC subfamilies are the most extensively studied, with more than 50 chemokines and 20 receptors identified. The promiscuity of ligand–receptor interactions, where one chemokine can bind to several receptors or vice versa, further complicates their functional dynamics [17, 18].

Recent findings highlight the diagnostic and therapeutic potential of chemokines in TB [19, 20]. For example, interferon‐induced protein 10 (IP‐10/CXCL10) mediates the migration and activation of immune cells by binding to target cell receptors and plays a crucial role in TB defense [3]. Additionally, CXCL10 and CCL1 exhibit different expression levels in active TB compared to latent infection, making them promising biomarkers. However, due to small sample sizes in existing studies and population variability, further validation of the diagnostic value of chemokines with larger multicenter studies is still needed.

Furthermore, targeting chemokine signaling pathways, such as using CCR5 antagonists or modulating CXCR3, could improve TB treatment. These approaches may enhance granuloma function or reduce harmful immune responses [21]. However, understanding chemokine function at different stages of TB is still incomplete. Further research is needed to investigate their spatiotemporal regulation and interactions with host genetics [22].

Additionally, animal models can only partially reproduce chemokine responses in humans during Mtb infection. Although studies in mice provide valuable insights into chemokine dynamics, differences in immune system complexity, granuloma formation, and chemokine–receptor interactions between species may limit their applicability in translational study [23]. Humanized NSG‐SGM3 mice can recapitulate the pathogenic effects of HIV and Mtb infection and co‐infection at pathological, immunological, and metabolic levels, making them a reproducible small animal model for studying HIV/Mtb co‐infection [24]. Future research should focus on cohort studies in humans and advanced *in vitro* models, such as organoids, to validate results and address this gap, ensuring clinical relevance for diagnosis and therapy.

**Fig. 1:**
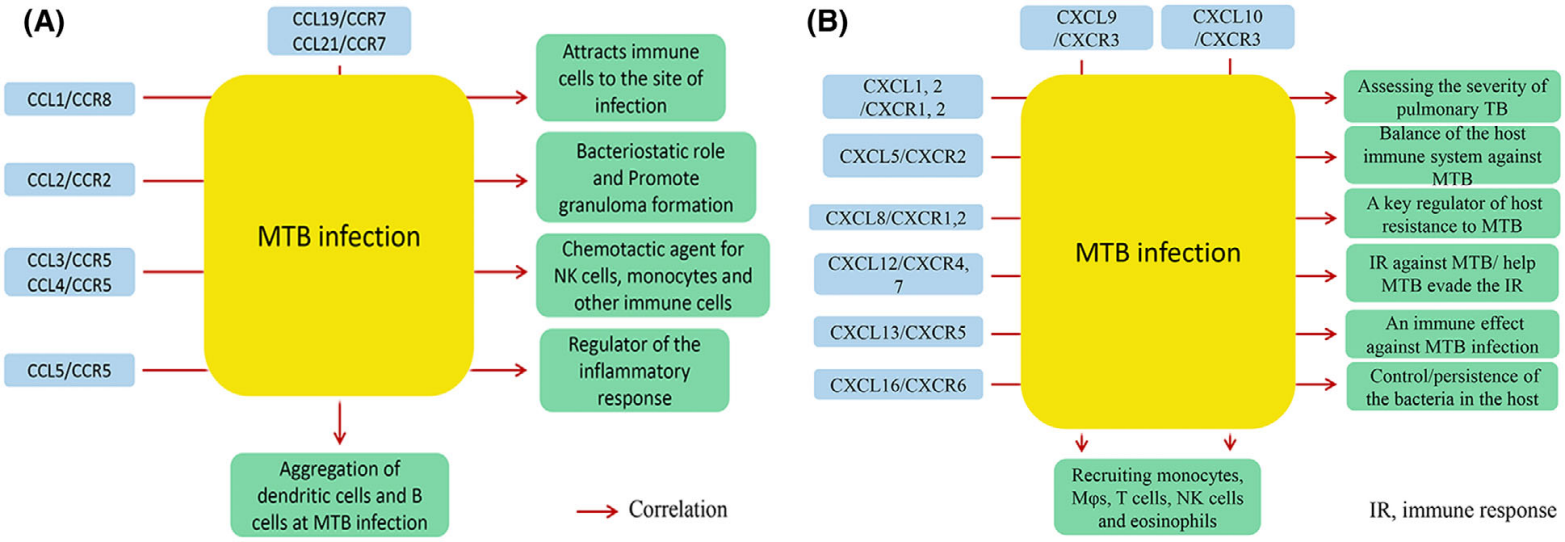
Chemokines and their receptor pathways involved in Mtb infection. The role of CC chemokines and their receptors (A), and CXC chemokines and their receptors (B) in *Mycobacterium tuberculosis* (Mtb) infection. The functions of chemokines and their receptors are mostly indicated by the corresponding arrows (→). Chemokines and receptors often have overlapping functions, depending on the context.

Regarding chemokine‐based diagnostics, this review also summarizes multiplex assays and machine learning (ML), and critically evaluates their translational potential [25]. The chemokines and their receptors discussed in this review are listed in Table S1. Their characteristic parameters and functions are summarized in Table 1, and their pathways related to TB infection are shown in Fig. 1.

**Table 1:** Characteristic parameters and functions of chemokines.

Chemokines	Size (aa)	Expression location	Cellular sources	Functions	References
CCL1	96	Lymphoid tissues, activated T cells	Activated T cells, monocytes, endothelial cells	(1) Attracts monocytes, T cells, and DC (2) Involved in inflammatory and immune responses	[27]
CCL2	76	Lung, liver, kidney, etc. during inflammation	Monocytes, Mφ, endothelial cells, fibroblasts, smooth muscle cells	(1) Recruits monocytes, memory T cells, and DC to sites of inflammation	[32]
CCL3	70	Inflamed tissues, lymph nodes	Mφ, DC, T cells, mast cells	(1) Promotes recruitment of monocytes, lymphocytes, and neutrophils (2) Induces inflammatory cytokines	[37]
CCL4	69	Inflamed tissues, lymphoid organs	Mφ, DC, T cells	(1) Attracts monocytes, NK cells, and T cells (2) Synergizes with CCL3	[37]
CCL5	68	Inflamed tissues, lymphoid organs	T cells, platelets, Mφ, endothelial cells	(1) Strong chemoattractant for T cells, eosinophils, and basophils (2) Involved in allergic and inflammatory responses	[41]
CCL7	99	Uterus, heart, central nervous system, lung, retina, vertebral axis musculature	Activated macrophages, mast cells	(1) Attracts monocytes and eosinophils (2) Augments monocyte antitumor activity and is involved in inflammatory responses and metastasis	[34]
CCL12	104	Thymus, lymph nodes, bladder, genital fat pad, and various other tissues	Activated macrophages and mast cells under inflammatory conditions	(1) A potent chemoattractant for monocytes and lymphocytes (2) Plays a role in inflammatory responses and is involved in processes like lung fibrosis and allergic inflammation	[36]
CCL19	98	Lymph nodes, thymus, spleen	Stromal cells, DC	(1) Directs migration of naive T cells and DC to lymph nodes	[47]
CCL21	134	High endothelial Venules, lymphatic vessels	Endothelial cells, stromal cells	(1) Guides T cells and DC into lymph nodes and Peyer's patches	[49]
CXCL1	73	Especially in inflamed tissues	Mφ, neutrophils, fibroblasts, epithelial cells	(1) Neutrophil chemotaxis, angiogenesis	[52]
CXCL2	72	Especially in inflamed tissues	Mφ, neutrophils, fibroblasts, epithelial cells	(1) Neutrophil chemotaxis	[53]
CXCL5	93	Expressed in the central nervous system, liver, and sensory organs	Alveolar epithelial type II cells, eosinophils	(1) Neutrophil chemoattractant (2) Involved in inflammatory responses and host defense against bacteria	[54]
CXCL6	77	Biased expression in spleen, gall bladder, and other tissues	Epithelial cells, macrophages, and mesenchymal cells	(1) Potent neutrophil chemoattractant (2) Antibacterial (3) Angiogenic	[55]
CXCL7	70	Not available	Activated platelets	(1) Neutrophil activator (2) Cell function modulator	[56]
CXCL8	72	Inflamed tissues	Mφ, neutrophils, endothelial cells, fibroblasts	(1) Neutrophil chemotaxis (2) Angiogenesis (3) T cell recruitment	[58]
CXCL9	103	Inflamed tissues, lymphoid organs	Activated T cells, Mφ, DC	(1) T cell chemotaxis (2) Th1 response	[69]
CXCL10	98	Inflamed tissues, lymphoid organs	Activated T cells, Mφ, fibroblasts, endothelial cells	(1) T cell chemotaxis (2) Th1 response (3) Antiangiogenic	[64]
CXCL11	94	Inflamed tissues, lymphoid organs	Activated T cells, Mφ, DC	(1) T cell chemotaxis (2) Th1 response	[65]
CXCL12	89	Especially in bone marrow, lymph nodes	Stromal cells, endothelial cells, fibroblasts	(1) Stem cell homing (2) lymphocyte trafficking (3) Angiogenesis	[71]
CXCL13	103	Lymphoid tissues, inflamed tissues	Follicular DC, Mφ	(1) B‐cell chemotaxis3 (2) Follicle formation	[76]
CXCL16	254	Especially in Mφs, DC	Mφ, DC, endothelial cells	(1) T‐cell chemotaxis, adhesion (2) Scavenger receptor function	[79]

*Note:* This table provides a general overview and is not exhaustive. Expression location and cellular sources may vary by context. Chemokines often have overlapping functions and may interact with multiple receptors. Only one representative reference is listed in the table; additional relevant references are available in the corresponding sections of the main text. 1. Size (aa): Number of amino acids in the mature chemokine protein. 2. Expression location: Indicates where the chemokine is typically expressed, either constitutively or during inflammation. 3. Cellular sources: Lists the primary cells responsible for synthesizing the chemokine. 4. Functions: Describes the main biological functions, especially in recruiting and transporting immune cells.

Abbreviations: DC, dendritic cells; Mφ, Macrophage.

## CC/CXC chemokines and receptors

### CCL1

CCL1 is a chemokine from the CC chemokine family, and its receptor is CCR8 [26]. It is secreted by activated T cells and attracts immune cells such as monocytes, natural killer (NK) cells, and dendritic cells (DCs) to infection sites [27]. High expression of CCL1 indicates a sustained immune response against Mtb. The concentration of CCL1 is significantly increased in the plasma of patients with pulmonary tuberculosis (PTB) [28]. Additionally, the expression levels of CCL1 and interleukin‐2 receptor alpha (IL‐2Ra; CD25) are higher in TB patients than in latently infected individuals, and these markers have significant potential for differential diagnosis between the groups [29]. In distinguishing between non‐HIV‐infected individuals and those with active pediatric TB or LTBI, plasma CCL1 is among the 20 most accurate diagnostic marker combinations, including CCL1, CCL5, CRP, and MIP‐1α. These four biological diagnostic indicators can distinguish active TB from LTBI independently of HIV infection [30].

### CCL2, CCL7, and CCL12

The chemokines CCL2, CCL7, and CCL12 play a central role in the immune response to Mtb infection. CCL2, also known as monocyte chemoattractant protein‐1 (MCP‐1), is a potent proinflammatory chemokine in the CC family, and its receptor is CCR2 [31]. A study shows that CCR2 has a novel protective role in TB immunity by promoting the migration and functional transition of alveolar macrophages from the airways into granulomas during infection of mice with the clinically relevant HN878 Mtb strain [32]. Moreover, in a mouse model, CCL2 promotes macrophage migration, phagocytosis, and effector molecule expression, as demonstrated by recombinant BCG expressing CCL2 (rBCG‐CCL2), which induces robust innate and adaptive immune responses in the lung and reduces bacterial burden and pathology [33]. CCL7, also known as MCP‐3, was first cloned and characterized from the supernatant of human osteosarcoma cells [34]. Elevated plasma levels of CCL2 (MCP‐1) and CCL7 (MCP‐3) are found in both drug‐sensitive and multidrug‐resistant TB patients and correlate with differences in leukocyte migration and chemotaxis [35]. Along with CCL2, CCL12 is also upregulated by MASP‐2 in BCG‐infected mice, promoting the recruitment of macrophages and lymphocytes into granulomas and increasing cytokine production (e.g., IFN‐γ, TNF‐α), thereby enhancing bacterial defense [36]. These findings underscore the key role of chemokines in protective immunity and immunopathology in TB.

### CCL3 and CCL4

Human CCL3 and CCL4 are expressed in most mature hematopoietic cells, and 68% of their amino acids are homologous [37]. They share the receptors CCR1 and CCR5. CCL4 acts as a chemotactic agent for NK cells, monocytes, and other immune cells migrating to sites of infection. It induces cytokines such as IL‐1, IL‐6, and TNF‐α by stimulating their synthesis and release from fibroblasts and macrophages. CCL3 levels in saliva and serum have been considered a distinguishing feature between TB and other human lung diseases [38]. However, Kizza et al. showed that the specificity of CCL4 is not high when using cross‐flow analysis to determine CCL4 concentration in pleural effusion and whole blood for diagnosing human TB, which requires further research [39]. One study performed univariate and multivariate conditional regression analyses of chemokines in the plasma of TB patients and found that baseline levels of CCL3 and CCL4 were associated with an increased rate of unfavorable treatment outcomes. The study also found that the sensitivity and specificity of CCL4 in plasma were low for diagnosing pulmonary TB compared to CCL3, suggesting that CCL3 is more promising as a biological indicator for predicting TB prognosis [12, 40].

### CCL5

CCL5 was the first highly expressed secreted protein identified in human T cells infected with HIV‐1. Because it is expressed after T‐cell activation, it was also named Regulated on Activation, Normal T‐cell Expressed and Secreted (RANTES) [37]. The receptors for CCL5 are CCR1 and CCR5, which are primarily found on macrophages, T cells, and dendritic cells. After binding to CCL5 and other ligands, CCR5 regulates the function of inflammatory cells and is an important regulator of the inflammatory response [41]. A study in a mouse model showed that Mtb can stimulate the expression of CCR5 in macrophages and induce the production of immunosuppressive cytokines through downstream signal transduction, impairing the immune response [42]. This suggests that CCR5 may increase the susceptibility of mice to TB. However, increased expression of CCR5 had no effect on the survival of Mtb in mouse macrophages, indicating the possibility of other immunoregulatory mechanisms. After infection with Mtb in CCR5‐deficient mice, the expression of pro‐inflammatory factors increases, which activates macrophages to exert bactericidal effects. Therefore, CCR5 deficiency plays an important role in the host immune response against Mtb [43]. A study using mouse whole genome mapping analysis showed that high levels of CCL5, interferon‐γ, and CXCL9 have a protective effect against Mtb infection, suggesting that CCL5 can promote the killing of Mtb by macrophages [44].

Although containment of Mtb within granulomas typically depends on chemokine‐mediated cellular recruitment, Algood and Flynn showed that CCR5‐deficient mice unexpectedly maintain an effective Th1 response and control infection, despite increased pulmonary lymphocytic infiltration, elevated inflammatory cytokine levels, and disrupted dendritic cell trafficking to lymph nodes [45]. This paradox underscores the pivotal role of CCL5 in TB pathogenesis. After stimulation with Ag85A, RANTES expression in mononuclear cells from the pleural fluid of patients with tuberculous pleurisy is lower than in nontuberculosis patients. Further research is needed to evaluate its potential as a diagnostic biomarker for pleural tuberculosis [46]. Thus, CCL5 not only orchestrates a critical immune response against Mtb but also shows a strong correlation with infection‐specific gene expression, making it a promising candidate for future TB diagnostic biomarkers.

### CCL19 and CCL21

CCL19 and CCL21 are key chemokines that regulate immune responses during Mtb infection, both acting through the shared receptor CCR7 [47]. Immunoplasma profiling studies have identified CCL19 as a crucial component of a nine‐protein biomarker signature that distinguishes active TB from healthy controls with high accuracy (AUROC 0.89–0.99), highlighting its diagnostic potential [48]. CCL21, produced by fibroblastic reticular cells (FRCs), guides dendritic cells (DCs) and T cells into lymph nodes (LNs). In chronic TB, abnormal B‐cell accumulation disrupts LN architecture, disperses the CCL21‐producing FRC network, and impairs T‐cell activation in mouse models [49]. Additionally, while Mtb‐infected DCs rely on HIF1A‐driven glycolysis for CCL21‐directed migration, monocytes from TB patients show impaired glycolysis. This metabolic defect hinders DC trafficking and delays effective immune response [50]. These findings underscore the importance of CCL19 and CCL21 as regulators of immune cell trafficking and lymph node integrity in TB, suggesting distinct clinical applications: using CCL19 for diagnostics and developing host‐targeted therapies to address CCL21‐mediated migration defects.

### CXCL1, CXCL2, CXCL5, CXCL6, and CXCL7

While neutrophils are the primary reservoir for CXCR1 and CXCR2, these receptors are also found on NK cells, T cells, and monocytes [51]. They act as molecular anchors for a specific group of chemokines—CXCL1, CXCL2, CXCL5, CXCL6, and CXCL7, which coordinate the recruitment and activation of neutrophils. This places neutrophils at the center of a double‐edged sword: they are essential for protective immunity but can also drive immunopathology [52]. In mouse models, CXCR2 mediates the influx of CD101‐negative neutrophils, a process associated with interferon‐mediated tissue damage and impaired bacterial control [53]. Elevated levels of CXCL5 and CXCL6 alter leukocyte migration patterns [54], which are associated with distinct changes in monocyte and T‐cell subsets in patients with multidrug‐resistant TB (MDR‐TB) [35]. Although CXCL6 typically regulates pulmonary T cell responses through CXCR1/2 signaling, its absence in mouse models paradoxically improves Mtb control by fine‐tuning inflammatory kinetics without impairing T cell recruitment [55]. Khajoee et al. suggest that in mice, CXCL7 and osteopontin may contribute to host resistance to mycobacteria [56]. These findings highlight the dual role of CXCL chemokines in TB: they are essential for enhancing neutrophil and T‐cell defenses, but also exacerbate inflammatory pathology.

### CXCL8

CXCL8 (IL‐8) is a key orchestrator in the human immune system, responsible for recruiting and activating various immune cells [57]. In patients with nontuberculous mycobacterial pulmonary disease (NTM‐PD), the CXCL8‐CXCR1/2 axis (the murine homolog of CXCL1/2) is significantly upregulated, promoting the expansion of IFIT1+ neutrophil subclusters and enhancing their interactions with mononuclear phagocytes and NK cells [58]. Evidence also supports its role in the structure of TB granulomas, where it acts as a master regulator of host resistance against Mtb [59]. Specifically, CXCL8 strengthens the body's defense against human TB by recruiting neutrophils to the infection site [60]. Additionally, a combined plasma signature of CCL3, CXCL8, and CXCL10 is strongly associated with a higher risk of poor treatment outcomes in the PTB patient validation cohort. In both the test cohort (*n* = 201) and the validation cohort (*n* = 60), the area under the curve (AUC) for this chemokine combination ranged from 0.937 to 0.969, with sensitivity between 82% and 96% and specificity between 87% and 92%. Therefore, Kumar et al. [12] suggest that plasma CXCL8 is a strong predictive biomarker for PTB treatment efficacy. This is supported by S Selvavinayagam et al. [61], who identified CXCL8 and MCP‐1 as surrogate biomarkers for patients with latent tuberculosis infection (LTBI), particularly in resource‐limited settings. Thus, CXCL8 is a promising marker for both the diagnosis and prognosis of TB. Moreover, targeting key signaling pathways such as the CXCL8‐CXCR1/2 or CXCR6 axes may offer new therapeutic strategies for TB and related mycobacterial infections.

### CXCL9, CXCL10, CXCL11

CXCL9 (MIG), CXCL10 (IP‐10), and CXCL11 (I‐TAC) coordinate immune responses through their shared receptor, CXCR3 [62]. These chemokines are essential for Th1 cell function, mobilizing immune effectors, such as monocytes, macrophages, T cells, NK cells, and eosinophils. Along with CXCR3, this group forms a key component of the host response in patients with TB [63], specifically directing CXCR3+ Th1 cells to sites of infection. Paradoxically, Mtb undermines this protective mechanism by manipulating host tryptophan metabolism. By upregulating indoleamine 2,3‐dioxygenase 1 (IDO1) in mouse models, Mtb increases kynurenine (Kyn) levels, which activate the aryl hydrocarbon receptor (AhR). This pathway suppresses STAT1 signaling, reduces secretion of CXCL9 and CXCL10, and delays T cell recruitment, promoting bacterial persistence [64]. In contrast, elevated levels of CXCL9 and CXCL10 are characteristic of pulmonary, tubercular, and drug‐resistant TB; these levels correlate with disease severity and have significant diagnostic value. Notably, CXCL10 and CXCL9 can distinguish drug‐resistant from drug‐susceptible TB patients with high accuracy [65]. Additionally, in patients with PTB, interleukin‐27 (IL‐27) modulates CXCL10 expression within a complex regulatory network involving miRNAs such as hsa‐let‐7b‐5p and hsa‐miR‐30a‐3p [66]. Thus, CXCL9, CXCL10, and CXCL11 act as double‐edged swords: while essential for protective immunity, they are also exploited by Mtb to evade immune surveillance. Targeting their regulatory pathways, particularly through CXCR3, may reveal new therapeutic strategies for TB.

In a meta‐analysis of 18 studies involving 2838 TB patients, Qiu et al. [67] evaluated the diagnostic performance of CXCL10, reporting a sensitivity of 86% and specificity of 88% for detecting PTB, effectively distinguishing TB from non‐TB cases. Supporting these findings, Wang et al. [68] experimentally confirmed that CXCR3 and its ligands are strong markers for active PTB in patients, with CXCL11 and CXCL9 playing key roles in clinical evaluation. Therefore, the CXCR3 axis and its ligands are promising biomarkers for diagnosing TB and assessing disease severity in humans [69], although heterogeneity and generalizability should be validated in future studies.

### CXCL12

Chemokine ligand 12 (CXCL12), also known as stromal cell‐derived factor‐1 (SDF‐1), plays a pivotal role in the pathogenesis of Mtb [70]. As a chemotactic signal, CXCL12 recruits T cells and dendritic cells to sites of infection or tissue injury through the CXCR4 or CXCR7 receptors, facilitating granuloma formation—structures essential for mounting an immune defense against Mtb [71]. Paradoxically, Mtb subverts this protective mechanism by upregulating CXCL12 secretion; this increase drives the aggregation of immune cells into granulomas, which the pathogen exploits as a sanctuary for persistence. Within these granulomatous niches, virulent Mtb strains in the zebrafish model induce host miR‐206 expression, silencing the CXCL12/CXCR4 signaling axis and blocking recruitment of protective neutrophils [72]. By promoting granuloma formation while suppressing effector immune responses, Mtb maintains a balance between containment and evasion, sustaining chronic infection [73]. Additionally, evidence indicates that CXCL12 is more useful as a prognostic marker than as a diagnostic tool for active human TB [74]. The multifaceted role of CXCL12 in Mtb infection highlights the complex interactions between host and pathogen in human TB.

### CXCL13

CXCL13, also known as B‐cell‐attracting chemokine 1 (BCA‐1) [75], acts as a selective chemoattractant for B‐1 and B‐2 cell subsets by binding to the CXCR5 receptor [76]. Although its diagnostic and prognostic value in PTB is still under investigation, evidence from murine models shows that Mtb infection induces coordinated upregulation of both CXCL13 and CXCR5, orchestrating a robust immune response [15]. Recent studies indicate that serum detection of CXCL13 together with IP‐10 can effectively distinguish active human TB from latent TB infection (LTBI), with an area under the curve (AUC) of 0.83 [77]. While CXCL13 is not yet an established clinical marker, it has significant research potential; for example, Ardain et al. showed that Group 3 innate lymphoid cells (ILC3s) play a key protective role in early pulmonary TB. In this context, Mtb‐induced CXCL13 upregulation recruits ILC3s to lung lesions via CXCR5, leading to the secretion of IL‐17 and IL‐22, which promote granuloma formation within lymphoid follicles and enhance early anti‐TB activity in mouse models [15]. These findings highlight how the chemokine–receptor interaction serves as a molecular beacon, guiding T cells to infection sites in mouse models—a mechanism that clarifies TB pathogenesis and offers promising opportunities for therapeutic intervention [78].

### CXCL16

CXCL16, also known as SCYB16 (Small‐inducible Cytokine B16), is a multifunctional chemokine that orchestrates the immune response against Mtb [79]. Upon infection, CXCL16 is upregulated in various immune effectors, including macrophages, dendritic cells, and T cells, where it interacts with its receptor CXCR6 on myeloid and T‐cell populations. This ligand–receptor interaction enhances macrophage‐mediated phagocytosis and bacterial clearance, underscoring its key role in innate immunity by limiting microbial replication in mouse models [55]. However, transcriptome profiling of Mtb antigen‐stimulated PBMCs identifies CXCL16 among 10 significantly downregulated genes, indicating a complex mechanism of immune modulation [79]. While these findings suggest a protective function, the precise role of CXCL16 in TB pathogenesis and its potential as a therapeutic target requires further investigation.

Paradoxically, recent evidence suggests that CXCL16 may also promote Mtb persistence in patients. It contributes to the formation of intracellular sanctuaries, known as latent TB granulomas, where Mtb adopts a dormant phenotype to evade immune surveillance in TB patients [80]. Mo et al. used single‐cell sequencing of pediatric tuberculous meningitis to identify a complement‐activated microglial population (Macro_C01) that recruits pathogenic Th17/Th1 CD4_C04 T cells through the CXCL16/CXCR6 signaling axis, thereby amplifying the neuroinflammatory cascade in the cerebrospinal fluid [81]. Thus, CXCL16 acts as a double‐edged sword in Mtb infections, exhibiting both protective and pathogenic properties. Understanding how CXCL16 modulates host–TB interactions is crucial, as this knowledge may lead to new therapeutic strategies for preventing and managing TB.

## Summary and outlook

### Application of chemokine in the diagnosis of TB

Chemokines from the CC and CXC subfamilies, along with their receptors, are pivotal in the pathogenesis, progression, and prognosis of TB [82]. Specifically, CXCR3 and its ligands are potential critical discriminators between active TB and latent infection, with expression changes detailed in Table S2. Recent animal model studies highlight CXCL1's diagnostic potential, demonstrating 94.5% sensitivity and 88.8% specificity in distinguishing active TB from latent TB infection (LTBI), as well as 90.9% sensitivity and 71.4% specificity compared to non‐TB cases [83]. Similarly, Gunasekaran et al. suggested that inflammatory mediators, including chemokines, play a crucial role in determining the outcome of Mtb infection and vary with disease severity, serving as potential diagnostic biomarkers for identifying latent tuberculosis reservoirs before symptoms appear [84]. Table 2 presents the sensitivity and specificity of representative chemokine biomarkers (CXCL10, CCL1, and CRP) in TB.

**Table 2:** Sensitivity/specificity of chemokine biomarkers (e.g., CXCL10, CCL1, CRP) in TB diagnosis.

Chemokines	Condition	Sensitivity (%)	Specificity (%)	AUC
CXCL10	Active TB vs. LTBI	86	88	0.83~0.93
	Active TB vs. Non‐TB	82~96	87~92	0.937~0.969
	Drug‐resistant TB vs. Drug‐sensitive	75	80	0.81
CCL1	Active TB vs. LTBI (HIV‐negative)	78	85	0.89
	Active TB vs. Controls	70	82	0.76
CRP	Active TB vs. LTBI	65~90	60~75	0.70~0.85
CCL19	Active TB vs. Controls	85	91	0.89~0.99
Combination (CCL1 + CXCL10 + CRP)	Active TB vs. LTBI	90	93	0.95

Abbreviations: AUC, Area under the curve; CRP, C‐reactive protein; LTBI, Latent tuberculosis infection.

The integration of machine learning with chemokine profiling represents a paradigm shift toward multiparameter diagnostic models. Using high‐dimensional data, this approach uncovers complex signatures that distinguish active TB, LTBI, and non‐TB conditions [85]. Researchers screened five potential serum protein biomarkers in a diverse outbred mouse population, with CXCL1 showing favorable performance in subsequent human sample validation. Bioinformatic analyses identified CCL2 and CXCL10 as key LTBI markers, each with AUC values above 0.85 [86]. High‐throughput proteomic platforms such as Olink and Luminex have validated CCL3's effectiveness in differentiating recent from remote infections [87]. Beyond diagnosis, machine learning links chemokine pathways to therapeutic targets, including connecting autophagy‐related genes such as FOXO1 to TB immunology [88].

Despite these advances, current findings are limited by small sample sizes, lack of external validation, and potential overfitting. Batch effects and cohort heterogeneity also compromise generalizability [89, 90]. Importantly, no prospective studies have validated these panels in high‐burden, resource‐limited settings, where cost, infrastructure, and assay standardization present significant barriers. Rigorous validation and implementation research are urgently needed before these tools can effectively augment conventional diagnostics in endemic regions.

### Chemokine in distinguishing stages of TB

Chemokine signatures show potential for differentiating the full spectrum of Mtb infection, from latent (LTBI) to subclinical and active TB (ATB). In selected cohort studies, CXCL10 and CCL1 demonstrate high diagnostic accuracy in distinguishing ATB from LTBI [29, 67], with CXCL10 achieving 86–88% sensitivity and specificity. Emerging evidence indicates that specific chemokines may identify subclinical TB, an intermediate state between LTBI and ATB, characterized by detectable bacterial activity without classic symptoms [11]. For example, one group developed a hierarchical method based on immune function to distinguish subclinical TB infection from other TB states [91]. Machine learning approaches that integrate multichemokine panels (such as CXCL1, CXCL2, TNF, and IL‐10) have improved discriminatory power across TB stages [83]. Validation of stage‐specific profiles, including CXCL13 for LTBI [77], combined with standardized high‐throughput assays, could enable precision diagnostics for comprehensive TB management, although this requires additional samples from multiple centers for verification.

Chemokine biomarkers may complement conventional TB diagnostics, including IGRA and PCR. One study reported high specificity in distinguishing active from latent infection, which could improve the interpretive accuracy of IGRA results [69]. Incorporating chemokine signatures such as CXCL9, CXCL10, and CCL19 into multiplex assays alongside PCR may enable rapid, noninvasive testing in resource‐limited settings [69]. Machine learning algorithms that analyze combined chemokine and conventional biomarker profiles show promise for predicting disease severity and treatment outcomes [48]. Studies have shown that CXCL1 is associated with a decreased risk of unfavorable outcomes in both unadjusted and adjusted analyses in the test cohort and is expected to become an immunological indicator for evaluating prognosis [12]. Additionally, CCL3, CXCL8, and CXCL10 are associated with an increased risk of unfavorable treatment outcomes in the validation cohort.

### Therapeutic implications of chemokine modulation in TB

Pharmacological modulation of chemokine networks offers a promising approach to recalibrate the balance between protective immunity and immunopathology in TB. The CXCR3–CXCL9/10/11 axis is crucial for Th1 cell recruitment, but inhibition may be necessary to limit excessive inflammation [92]. In human patients, CCR5 antagonists such as maraviroc have been investigated for their immunomodulatory potential in HIV and other infectious diseases, although blockade may impair antipathogen responses and alter cytokine profiles [42, 93, 94]. As shown in Table 3, each strategy presents distinct opportunities and challenges. For example, CCR2 inhibition may reduce monocyte‐driven inflammation but faces redundancy with other chemokine pathways. Clinical translation of these approaches is limited by the lack of large, well‐characterized cohort studies. Available data often come from small, heterogeneous populations with varying HIV co‐infection status and use different assay platforms (e.g., Luminex, Olink) without standardized cutoffs [95, 96]. These limitations impede validation of target safety and efficacy. Future research should prioritize prospective, multicenter studies with well‐defined cohorts to establish the therapeutic window and minimize off‐target effects before integrating these agents into host‐directed therapy regimens.

**Table 3:** Current strategies to inhibit CCR5, CXCR3, CCR2.

Feature	CCR5 inhibition	CXCR3 inhibition	CCR2 inhibition
Mechanism	Blocks CCR5 (a chemokine receptor used by HIV‐1 for entry), also involved in inflammatory cell migration	Inhibits CXCR3 (receptor for CXCL9/10/11), reducing Th1/T‐cell migration to inflamed tissues	Blocks CCR2, preventing monocyte/macrophage recruitment via CCL2 (MCP‐1)
Benefits	(1) HIV treatment/prevention (e.g., maraviroc) (2) Potential in autoimmune diseases (e.g., RA, MS)	(1) Reduces T‐cell‐mediated inflammation (e.g., psoriasis, IBD) (2) May attenuate organ transplant rejection	(1) Targets chronic inflammation (e.g., atherosclerosis, NASH) (2) Potential in neuroinflammation (e.g., Alzheimer's)
Risks/side effects	(1) Increased susceptibility to West Nile virus (2) Possible resistance in HIV (3) Off‐target immune effects	(1) Increased risk of infections (e.g., viral reactivation) (2) May impair antitumor immunity	(1) Potential for liver toxicity (2) May alter metabolic responses (3) Redundant pathways could limit efficacy
Clinical opportunities	(1) Cure strategies for HIV (e.g., gene editing with Δ32 mutation). (2) GVHD, inflammatory diseases	(1) Autoimmune diseases (e.g., lupus, vitiligo). (2) Fibrosis (e.g., lung, liver)	(1) Metabolic diseases (e.g., obesity, diabetes) (2) Fibrosis, cancer immunotherapy
Challenges	(1) Viral tropism shift to CXCR4 (2) Limited efficacy in late‐stage HIV	(1) Complex ligand/receptor interactions (2) Lack of selective inhibitors in trials	(1) Redundancy with CCR5/CCR1 (2) Poor CNS penetration for neuro diseases

### Potential adverse effects of selective chemokine targeting

Intervening in chemokine signaling cascades, as shown by CCR5 antagonism, carries inherent risks by disrupting normal immune mechanisms [42]. CCR5 is a critical mediator of immune effector trafficking, specifically recruiting monocytes, T cells, and dendritic cells to sites of infection or inflammation in humans. It also serves as a key co‐receptor for HIV‐1 entry into immune cells. Therapeutic strategies targeting CCR5, including gene editing, antibody blockade, and antagonists, offer promising antiretroviral therapy (ART)‐independent approaches to prevent or eliminate HIV‐1 disease progression [93]. Evidence shows that CCR5 deficiency can alter cytokine profiles, potentially exacerbating inflammatory responses or causing immune dysregulation [94]. Although CCR5 inhibitors show therapeutic promise for TB, their broad immunosuppressive effects require careful evaluation to balance clinical efficacy with the risk of increased susceptibility to opportunistic infections or immune‐mediated disorders.

### Challenges in clinical translation

Although CC and CXC chemokines show considerable promise for diagnosing and treating TB, their path to clinical application faces several anticipated barriers. The lack of standardized detection methods is a critical hurdle. Current research uses a heterogeneous array of platforms (such as Luminex, Olink, and ELISA), each with distinct sensitivity levels and cutoff thresholds; this variability impedes cross‐study comparisons and prevents the establishment of universal reference ranges [95,96]. Cost‐effectiveness is also a significant obstacle, especially in resource‐limited regions with the highest TB burden. While multiplex chemokine assays provide extensive data, they are financially prohibitive and require specialized infrastructure, unlike conventional diagnostics such as smear microscopy or IGRA [97]. Additionally, individual variability, influenced by host genetics, HIV co‐infection, and geographic differences, alters chemokine expression profiles and complicates biomarker validation [24]. For example, the specificity of CCL4 varies among populations, highlighting the need for validation in diverse cohorts, as observed in studies of other inflammatory conditions such as Sjögren's disease [98]. Addressing these challenges will require coordinated efforts to harmonize protocols, confirm findings through large‐scale multicenter studies, and develop affordable point‐of‐care solutions before chemokine‐based strategies can be integrated into routine TB management.

In conclusion, the complex role of chemokines in TB requires further investigation. Future research could focus on four key areas: first, identifying stage‐specific chemokine signatures to improve early diagnosis and disease surveillance; second, therapeutically modulating chemokine signaling to enhance immune defenses while reducing tissue damage, enabling new treatment strategies; third, applying knowledge of individual differences in chemokine dynamics to develop personalized medicine approaches for better outcomes; and fourth, designing chemokine‐based vaccines that precisely adjust immune responses to strengthen protection against TB. These strategies have the potential to transform diagnostics, therapy, and vaccine development for Mtb infection [84, 99].

## Conflict of interests

The authors declare no conflict of interest.

## Author contributions

XY and DX conceived and designed the project. XY and JY acquired, analyzed, and interpreted the data. XY, DX, and JY wrote and reviewed the paper.

## Supporting information


**Table S1.** Chemokine abbreviations, alternative names, and receptors.
**Table S2.** Chemokine expression changes between latent and active infection.

## Data Availability

The authors have nothing to report.
