# Supplementary material for: Emerging insights into CC and CXC chemokines and their receptors in Mycobacterium tuberculosis infection
Source: FEBS Open Bio. 2026 May 12;16(7):1244–58. doi: 10.1002/2211-5463.70269 (PMC13327060; doi:10.1002/2211-5463.70269)
Supplement: Supplementary file 1 — Table S1. Chemokine abbreviations, alternative names, and receptors. Table S2. Chemokine expression changes between latent and active infection. [file FEB4-16-1244-s001.docx]

**Supplementary Data**

# Table S1 Chemokine abbreviations, alternative names, and receptors

| Chemokine | Alternative names | Receptors |
| --- | --- | --- |
| CCL1 | I-309, SCYA1 | CCR8 |
| CCL2 | MCP-1, SCYA2 | CCR2, CCR4, ACKR1, ACKR2 |
| CCL3 | MIP-1α, SCYA3 | CCR1, CCR5 |
| CCL4 | MIP-1β, SCYA4 | CCR1, CCR5 |
| CCL5 | RANTES, MIP-5 | CCR1, CCR5 |
| CCL7 | MCP-3 | CCR1, CCR2, CCR3 |
| CCL12 | MCP-5 | CCR2 |
| CCL19 | MIP-3β, ELC | CCR7 |
| CCL21 | SLC | CCR7 |
| CXCL1 | GRO-α, MGSA, NAP-3, SCYB1 | CXCR2 |
| CXCL2 | GRO-β, MIP-2α, SCYB2 | CXCR2 |
| CXCL5 | ENA78, SCYB5 | CXCR2 |
| CXCL6 | GCP2, SCYB6 | CXCR1, CXCR2 |
| CXCL7 | β-TG, NAP-2, SCYB7 | CXCR2 |
| CXCL8 | IL-8, NAP-1, SCYB8 | CXCR1, CXCR2 |
| CXCL9 | MIG, SCYB9 | CXCR3 |
| CXCL10 | IP-10, CRG2, SCYB10 | CXCR3 |
| CXCL11 | ITAC, SCYB11 | CXCR3 |
| CXCL12 | SDF-1, PBSF, SCYB12 | CXCR4, CXCR7 |
| CXCL13 | BLC, BCA-1, SCYB13 | CXCR5 |
| CXCL16 | SR-PSOX, SCYB16 | CXCR6 |

Alternative names may vary depending on context, such as species or historical use, and some chemokines have more aliases than those listed. The names provided are among the most commonly used in the literature.

ACKR1, Atypical chemokine receptor1

SCYA1, Small-Inducible Cytokine A1

MCP-1, Monocyte chemoattractant protein-1

MIP-1α, Macrophage inflammatory protein- 1alpha

MIP-1β, Macrophage inflammatory protein-1beta

RANTES, Regulated on activation, normal T cell expressed and secreted

MIP-3β, Macrophage inflammatory protein- 3beta

ELC, EBI1 ligand chemokine

SLC, Secondary lymphoid tissue chemokines

GRO-α, Growth-regulated oncogene alpha

MGSA, Melanoma growth-stimulating activity

NAP-3, Neutrophil-activating protein-3

SCYB1, Small-inducible cytokine B1

GRO-β, Growth-regulated oncogene beta

MIP-2α, Macrophage Inflammatory Protein-2alpha

ENA78, Epithelial-derived Neutrophil-activating peptide 78

GCP2, Granulocyte Chemotactic Protein-2

β-TG, β-thromboglobulin

NAP-2, Neutrophil-activating peptide-2

IL-8, Interleukin-8

MIG, Monokine induced by interferon-gamma

IP-10, Interferon gamma-induced protein-10

CRG2, Controlled by Retinoic Acid Gene 2

ITAC, Interferon-inducible T-cell alpha chemoattractant

SDF-1, Stromal cell-derived factor-1

PBSF, Pre-B cell growth-stimulating factor

BLC, B Lymphocyte chemoattractant

BCA-1, B Cell-Attracting Chemokine-1

SR-PSOX, Scavenger receptor for phosphatidylserine and oxidized low density lipoprotein

# Table S2 Changes in chemokine expression between latent and active TB infection

| Chemokine* | Latent MTB infection | Active TB | References |
| --- | --- | --- | --- |
| CCL1 | → | ↑ | [28] |
| CCL2 | ↑ | ↑ ↑ | [32] |
| CCL3 | ↑ | ↑ ↑ | [38] |
| CCL4 | ↑ | ↑ ↑ | [40] |
| CCL5 | → | ↑ | [38] |
| CXCL1 | → | ↑ | [28] |
| CXCL2 | → | ↑ | [28] |
| CXCL8 | → | ↑ | [61] |
| CXCL9 | ↑ | ↑ ↑ | [67] |
| CXCL10 | → | ↑ | [67] |
| CXCL11 | ↑ | ↑ ↑ | [68] |
| CXCL12 | → | ↓ | [74] |
| CXCL13 | → | ↑ | [77] |
| CXCL16 | ↑ | ↑ ↑ | [69] |

The changes in the chemokines are comparable to those in healthy controls (HC).

* No relevant reports were found for CCL19 and CCL21

↓, Decreased in serum and BAL fluid;

↑, Increased in serum and BAL fluid;

↑↑, Further increased in serum and BAL fuild;

→, No significant change

BAL, bronchoalveolar lavage fluid
